# Root canal treatment of a six-canal first mandibular molar with extensive periapical lesion: A case report

**DOI:** 10.1097/MD.0000000000034336

**Published:** 2023-07-28

**Authors:** Xin Li, Shuyu Sun, Tengyi Zheng

**Affiliations:** a Department of Endodontics, Affiliated Stomatological Hospital of Southern Medical University, Guangzhou, Guangdong, China; b School of Pharmaceutical Sciences, Guangdong Pharmaceutical University, Guangzhou, Guangdong, China.

**Keywords:** anatomical variation, case report, extensive periapical lesion, first mandibular molar, root canal treatment

## Abstract

There is an increasing tendency for case reports to reveal anatomical aberrances in mandibular first molars, such as the lateral and accessory canals. Thus, clinicians should pay special attention to anatomic variances when dealing with mandibular first molars requiring endodontic treatment to prevent reinfection within the root canal system, which is responsible for the failure of root canal treatment.

**Patient concerns:**

This article presents the clinical report and successful root canal treatment of a 24-year-old healthy female patient with an extensive periapical lesion in a 6-canal first mandibular molar. The patient was admitted to the endodontic department because of a periapical abscess found 1 month ago in her left mandibular first molar.

**Diagnosis:**

Chronic apical periodontitis was diagnosed based on clinical examination coupled with radiographic and cone-beam computed tomography images.

**Interventions:**

The treatment plan was to first perform root canal therapy and then perform clinical observation.

**Outcomes:**

During 1-year follow-up period, the treated tooth was asymptomatic, and complete resolution of the extensive apical lesion was eventually achieved, as shown in the postoperative cone-beam computed tomography images and clinical examination.

**Lessons:**

The present case emphasizes the importance of a comprehensive understanding of root canal morphology, especially rare anatomical variations, to ensure successful root canal treatment. Additionally, the case report adds to the library of previously reported cases of extensive periapical lesions with a direct connection to the root canal system, which demonstrates the potential clinical advantages of root canal therapy as a conservative nonsurgical approach in these cases.

## 1. Introduction

Apical periodontitis occurs as a result of anaerobic polymicrobial infection within the root canal system, which leads to an inflammatory and immune reaction of periapical tissue, followed by destruction of the periodontal ligament and apical bone. It is well established that root canal therapy is the most clinically effective treatment for pulpitis and apical periodontitis to prevent and treat the inflammation and destruction of periradicular tissue by adequately eradicating microbial infection from the root canal system.^[1–3]^ In addition, it is worth noting that current conventional treatment options for extensive apical lesions range from root canal therapy with or without apical surgery to tooth extraction. As mentioned in several clinical studies, large periapical lesions with a direct connection to the root canal system may respond favorably to nonsurgical root canal treatment with sufficient infection control.^[4–7]^ Therefore, it is of great importance to develop thorough awareness and understanding of root canal morphology for efficient root canal disinfection and successful endodontic treatment.

Growing evidence suggests that a complex root canal morphology is typically observed in mandibular first molars, which normally have 2 canals in the mesial root and 1 or 2 canals in the distal root. Infrequently, an accessory canal between the mesio-lingual (ML) and mesio-buccal (MB) canals in the mesial root is referred to as the mesio-middle (MM) canal a reported incidence ranging from 0.26 % to 46.2 %.^[8–11]^ Moreover, the disto-middle (DM) canal is located between the disto-lingual (DL) and disto-buccal (DB) canals, which are known as the third canal within a distal root, with a prevalence rate of 0.2 % to 3 %.^[12,13]^ The presence of 3 mesial and 3 distal canals in the mandibular first molar is extremely rare.

In this clinical report, we describe our endodontic treatment of extensive periapical lesions in a mandibular first molar with 6 root canals, including 3 mesial canals and 3 distal canals, to provide new references and evidence for clinical diagnosis, treatment, and prognosis.

## 2. Case presentation

**Figure 1.**
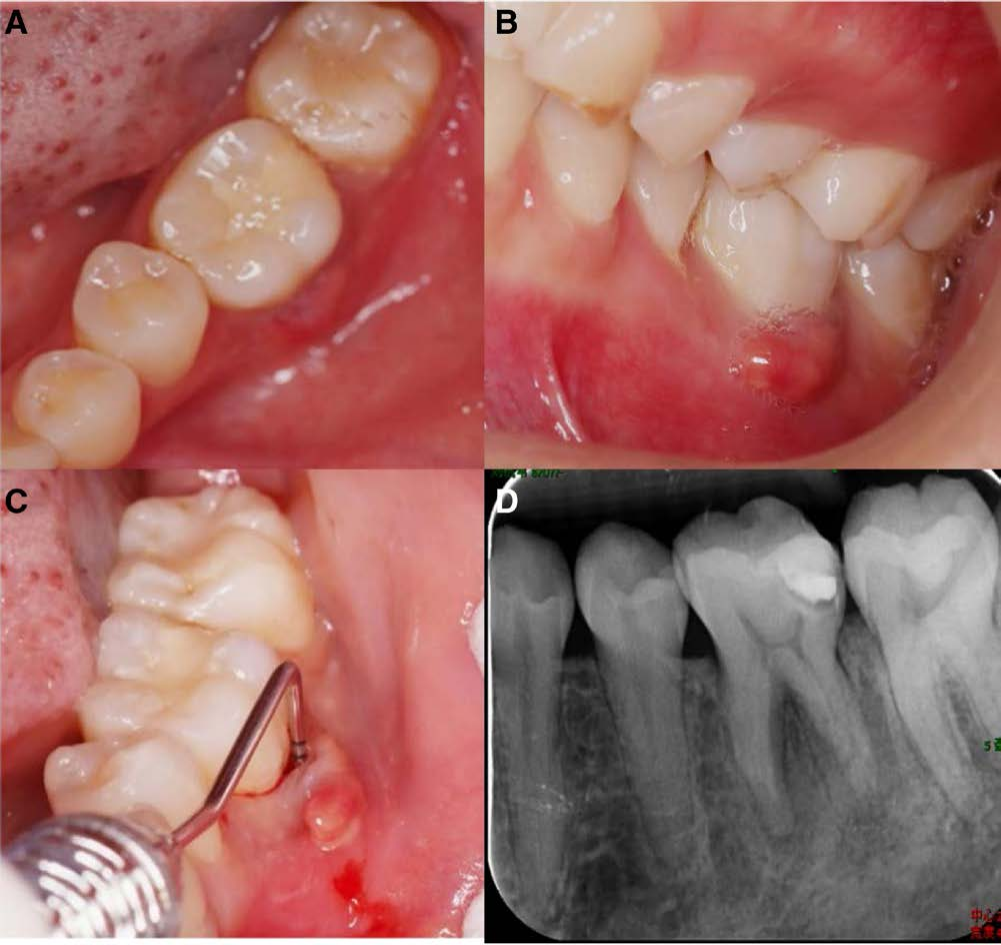
Preoperative photographs and radiographs of tooth 36. (A) A dental filling material in distal portion of occlusal and interproximal surface. (B–C) A fistula accompanied by severe periodontal disease (≥ 9 mm periodontal probing depth) in the buccal gingiva. (D) Extensive periapical lesion combined with severe inflammation-induced resorption of alveolar bone and mesial root.

A 24-year-old female patient was admitted to the endodontic department at the Affiliated Stomatological Hospital of Southern Medical University (Guangzhou, China) because of a periapical abscess found 1 month ago in her left mandibular first molar (tooth 36). The patient had no history of drug/food allergy or hereditary diseases in her family. On clinical examination, dental filling material was detected in the distal portion of the occlusal and proximal surfaces of tooth 36 (Fig. 1A). Tenderness on vertical percussion was elicited, but no tooth mobility was observed in tooth 36. In addition, a fistula with purulent fluid was found in the buccal gingiva, accompanied by severe periodontal disease (periodontal probing depth ≥ 9 mm), while other gingival attachments were normal (Fig. 1B and C). Meanwhile, the diseased tooth showed no electrical activity and responded negatively to the thermal and cold tests.

**Figure 2.**
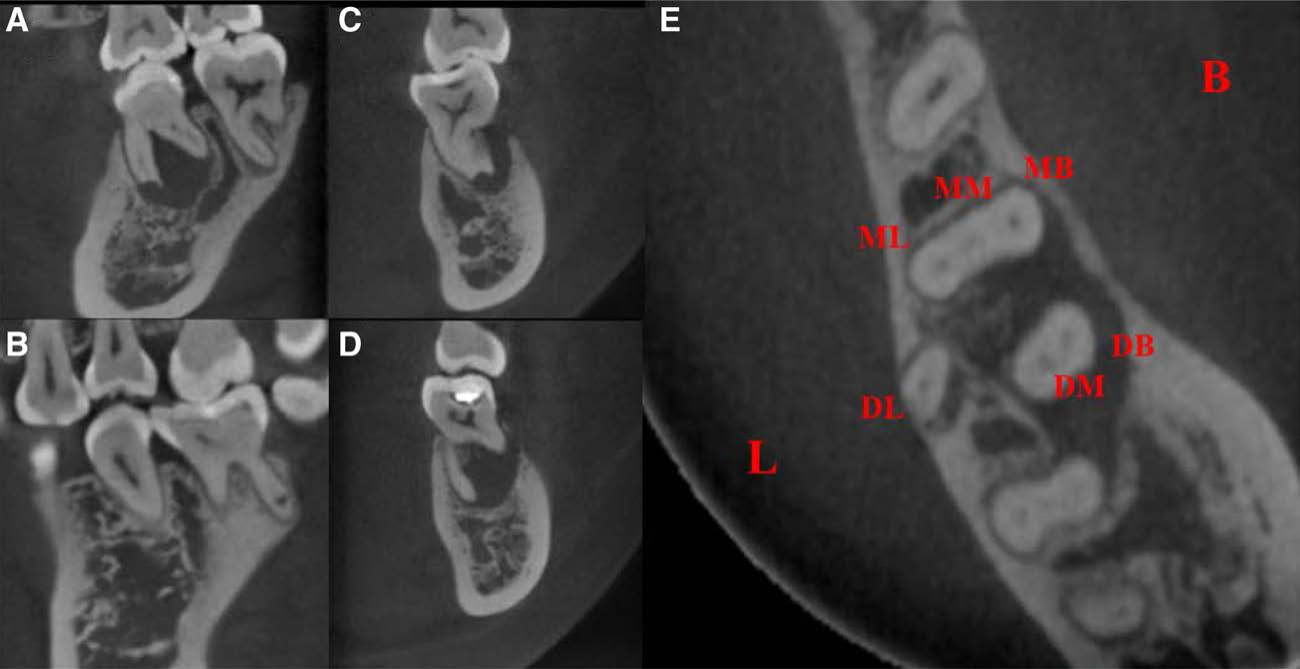
Preoperative CBCT scan of tooth 36. (A–B) Significant mesial and distal root resorption. (C–D) Apical lesion spreading to the mesial root of tooth 37 combined with alveolar bone resorption extending to the periapical area. (E) Three mesial (MB, ML and MM) and three distal (DL, DB and DM) orifices in the pulp chamber. CBCT = cone-beam computed tomography, DB = disto-buccal, DL = disto-lingual, DM = disto-middle, MB = mesio-buccal, ML = mesio-lingual, MM = mesio-middle.

Periapical radiographic examination showed the dental filling material in close proximity to the dental pulp and extensive apical lesion combined with severe inflammation-induced resorption of the alveolar bone and mesial root (Fig. 1D). Therefore, we applied cone-beam computed tomography (CBCT) for further periodontal examination, indicating alveolar bone resorption extending to the periapical area and significant mesial and distal root resorption in tooth 36, while a large apical lesion had spread to the mesial root of the left mandibular second molar (tooth 37) (Fig. 2A–D). In addition, cross-sectional CBCT analysis demonstrated that there were 3 mesial and 3 distal orifices in the pulp chamber (Fig. 2E). A tentative diagnosis of chronic apical periodontitis for tooth 36 was established based on clinical examination coupled with radiographic and CBCT images. The patient was given the option of root canal therapy or tooth extraction followed by a fixed prosthesis; however, the patient expressed a strong preference for endodontic treatment to retain her tooth. Therefore, the treatment plan was to first perform root canal therapy and then perform clinical observation.

**Figure 3.**
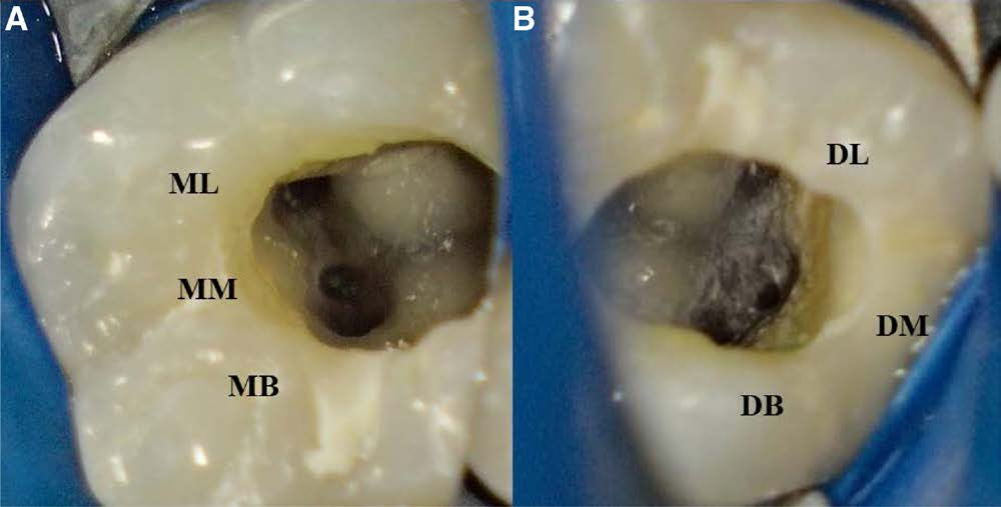
High-magnification view of six orifices in the pulp chamber of tooth 36. (A) Three mesial orifices. (B) Three distal orifices.

**Figure 4.**
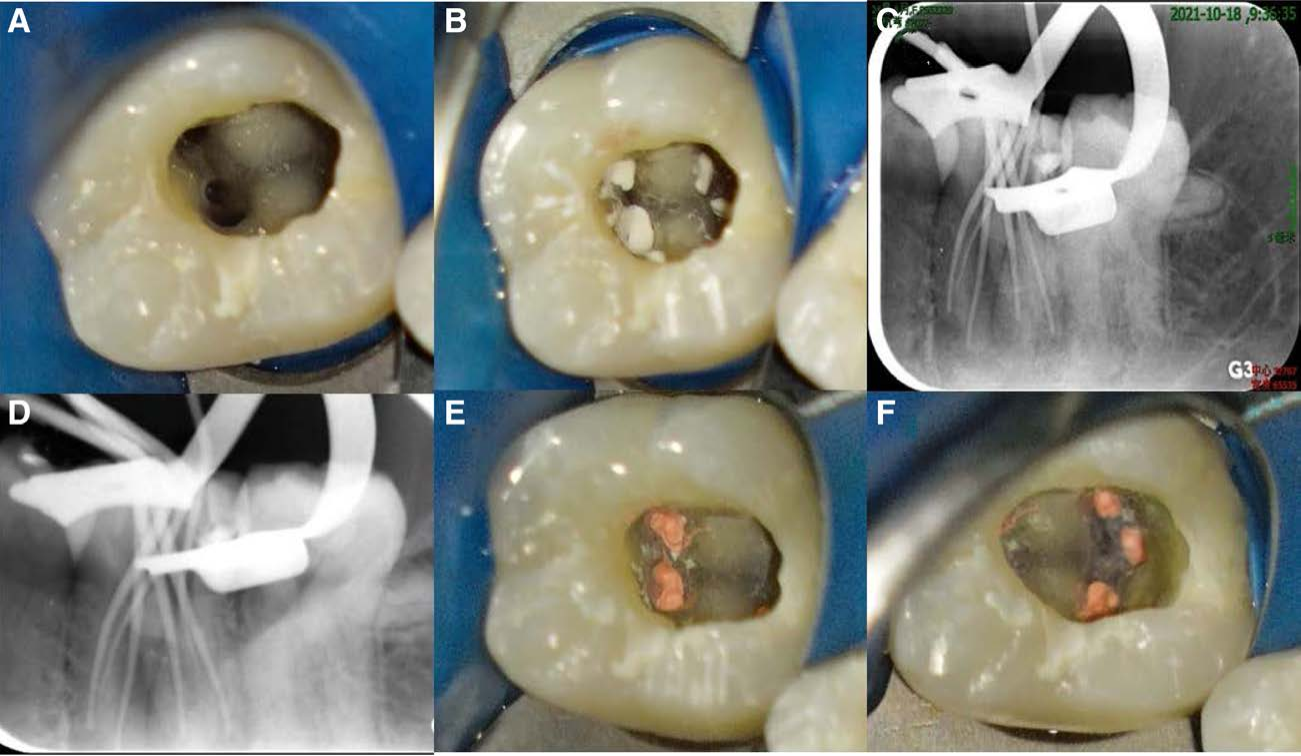
Root canal filling procedure of tooth 36. (A–B) Cleaning and filling the root canals with iRoot SP root canal sealer. (C–D) Periapical radiographs of gutta-percha cones in root canals. (E–F) Intraoral photographs of pulp chamber after root canal filling.

**Figure 5.**
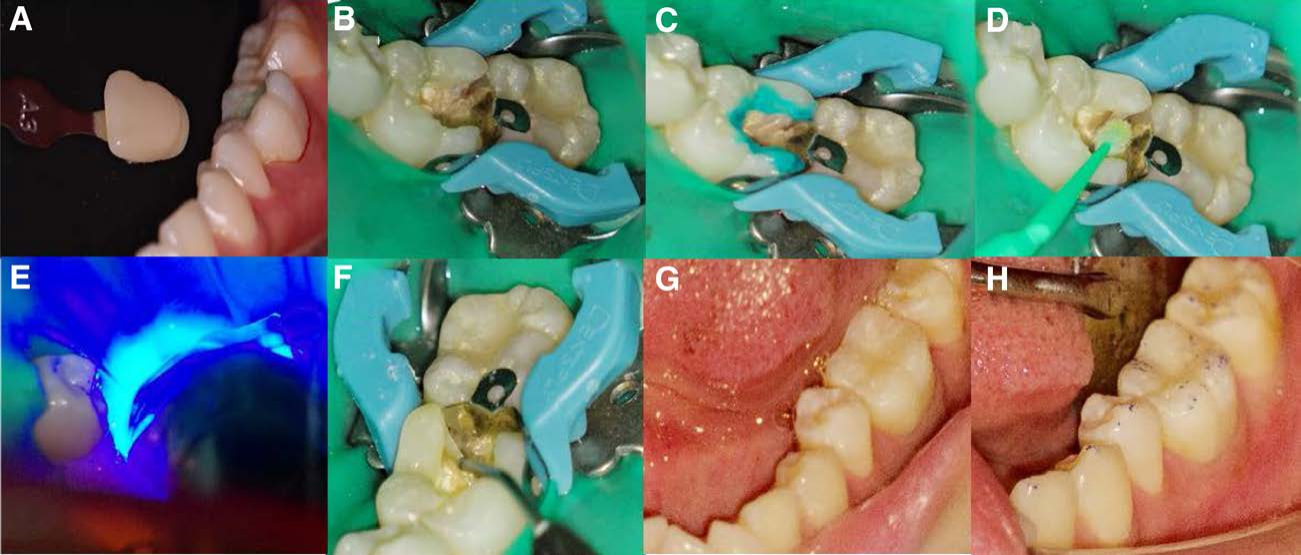
Composite resin restoration of the access cavity in tooth 36. (A) Dental shade matching. (B–D) Enamel acid etching and adhesive coating. (E) Light curing. (F–G) Flowable resin lining and composite resin restoration with sonic delivery technique. (H) Occlusal adjustment.

**Figure 6.**
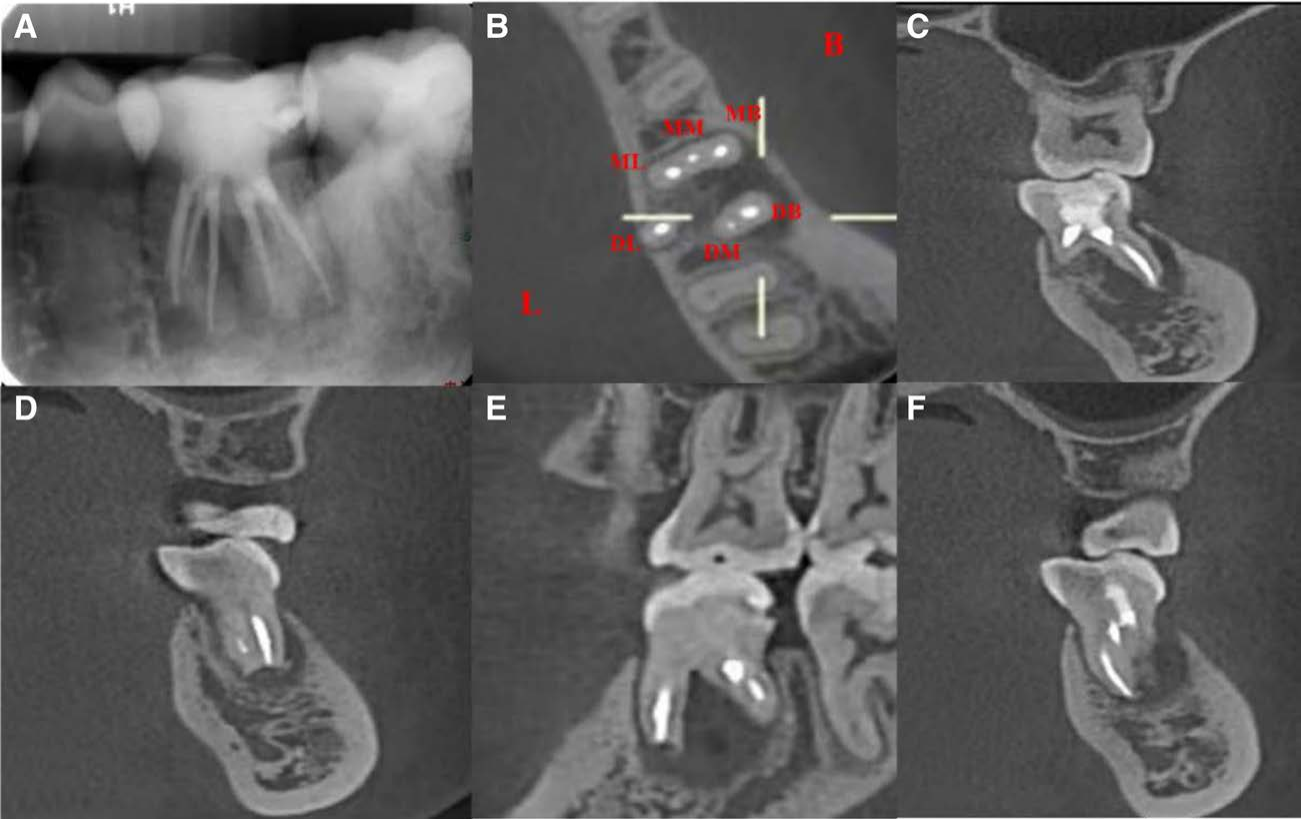
The immediate postoperative periapical radiographs and CBCT images of tooth 36. (A) Postoperative periapical radiographs. (B–F) Postoperative CBCT images. CBCT = cone-beam computed tomography.

**Figure 7.**
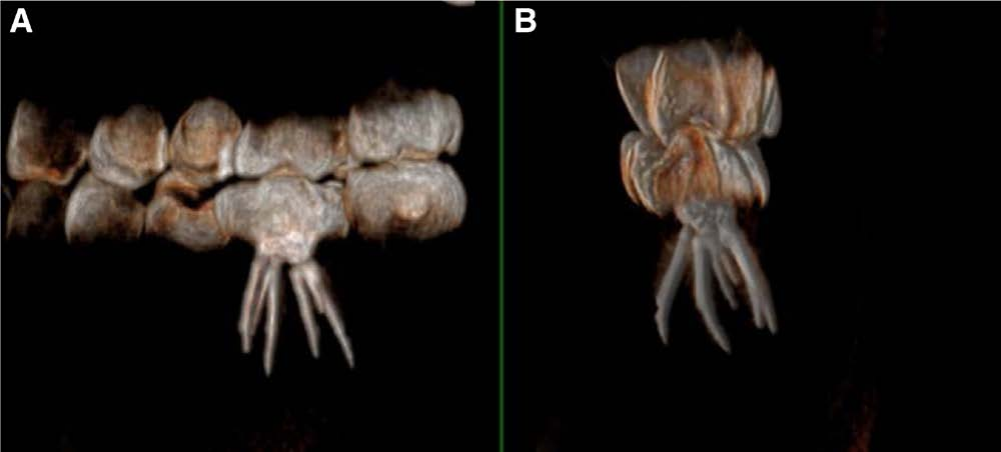
Three-dimensional reconstruction of the CBCT images of tooth 36. (A) Coronal view. (B) Sagittal view. CBCT = cone-beam computed tomography.

Treatment was initiated with the administration of inferior alveolar nerve block with 4 % articaine (Grand Pharma, Wuhan, China) and 1:100,000 epinephrine (Grand Pharma, Wuhan, China), followed by rubber dam isolation (Coltene, Altstatten, Switzerland). All subsequent procedures were performed using an operating microscope (Zeiss, Oberkochen, Germany). After removing the pulp chamber roof completely and irrigating the pulp chamber gently with 3 % sodium hypochlorite solution (Probita, Guangzhou, China), 6-canal orifices in tooth 36 were uncovered by exposure to microscope, which were identified as MB, ML, MM, DL, DB, and DM canals, respectively (Fig. 3). Following pulpectomy, the ML, MB, DL, and DB canals were negotiated to the working length, as confirmed by an electronic apex locator (VDW Dental, Munich, Germany), with a size 10 K-type file (VDW Dental, München, Germany). However, there are great difficulties in negotiating the MM and DM canals to the working length because of pulp calcification. After root canal preparation was completed using the WaveOne Gold nickel-titanium rotary file (Dentsply Sirona, York), all the canals were rinsed with 17 % ethylenediaminetetraacetic acid (Meta Biomed, Cheongju-si, Korea) and 3 % sodium hypochlorite solution alternately, and dried with sterile paper points. The access cavity was filled with calcium hydroxide paste (Ivoclar Vivadent AG, Schaan, Liechtenstein) for root canal disinfection and was temporarily sealed with glass ionomer cement (GC, Tokyo, Japan). At the second appointment after a week, the fistula healed completely, and tooth 36 was not sensitive to vertical percussion during routine oral examination. Following removal of the temporary filling and an alternating irrigation with 17 % ethylene diaminetetraacetic acid and 3 % sodium hypochlorite solution, a final rinse was carried out with normal saline and 2 % chlorhexidine (Probita, Guangzhou, China). Furthermore, a warm vertical compaction technique was employed to fill the root canals with gutta-percha points (Dentsply Sirona, York) and an iRoot SP root canal sealer (IBIOCERAMIX, Burnaby, Canada) (Fig. 4). After root canal obturation, the access cavity was restored with a composite resin restorative material (KERR, Orange) (Fig. 5), and postoperative apical radiographs and CBCT images were taken immediately (Figs. 6 and 7).

**Figure 8.**
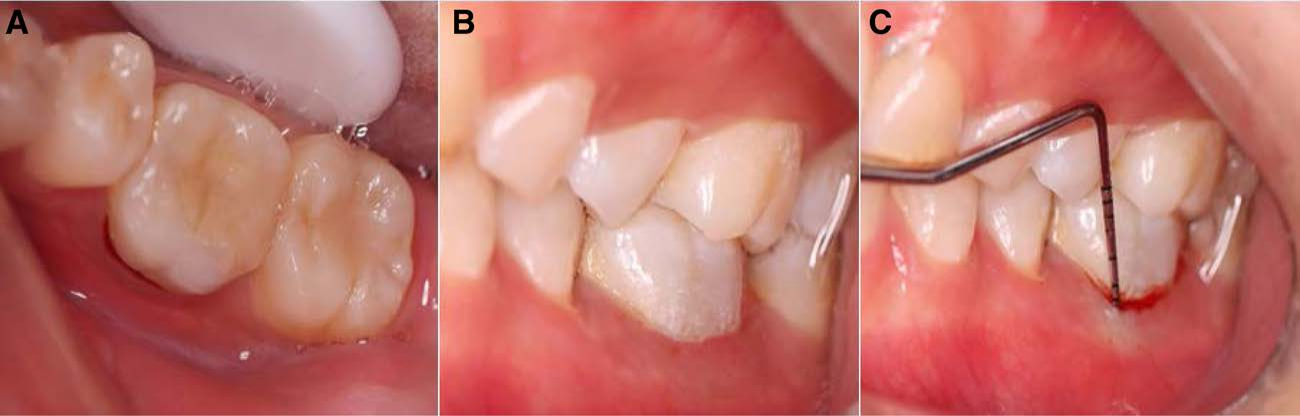
Intraoral photographs of tooth 36 at the 3-month follow-up visit. (A–B) NO fistulae in the buccal gingiva. (C) Periodontal probing depth within normal physiological limit.

**Figure 9.**
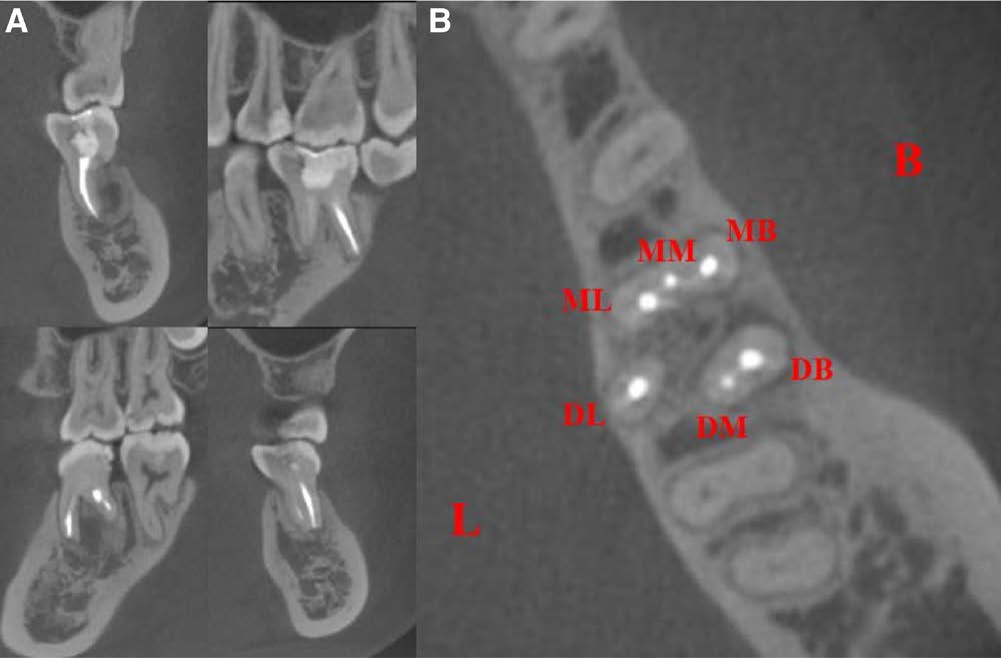
CBCT images of tooth 36 at the 3-month follow-up visit. (A) A significantly decreased apical lesion. (B) Cross-sectional view. CBCT = cone-beam computed tomography.

**Figure 10.**
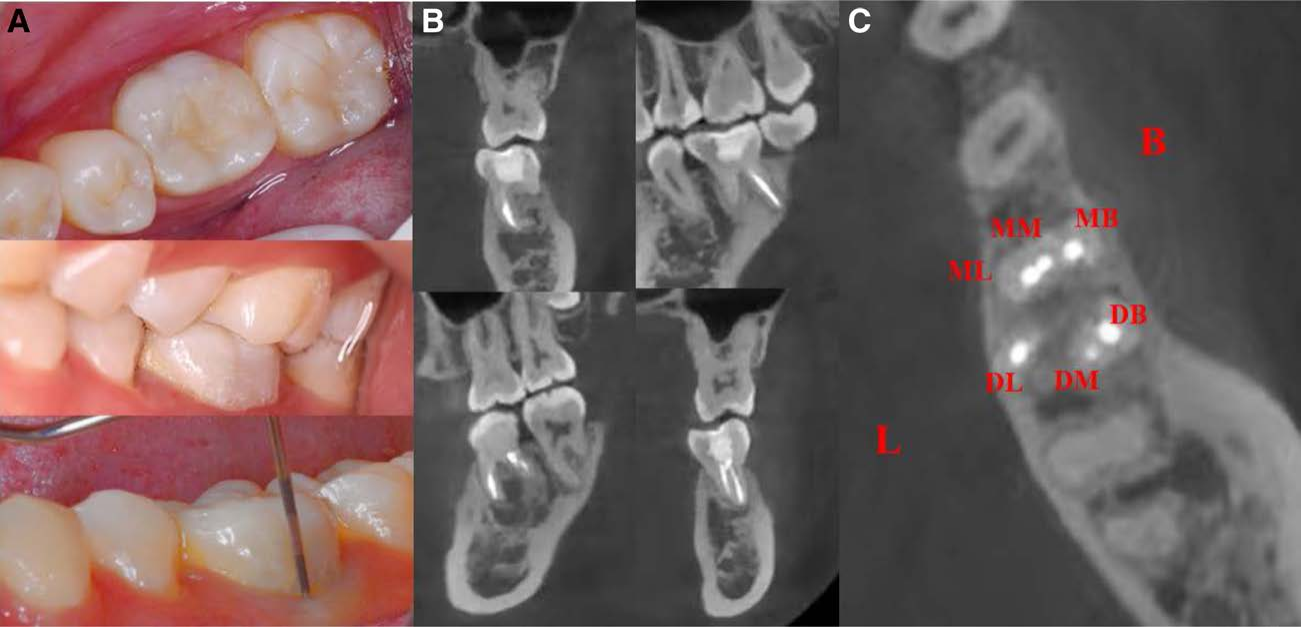
Intraoral photographs and CBCT images of tooth 36 at the 6-month follow-up visit. (A) Intraoral photographs, (B–C) CBCT images. CBCT = cone-beam computed tomography.

**Figure 11.**
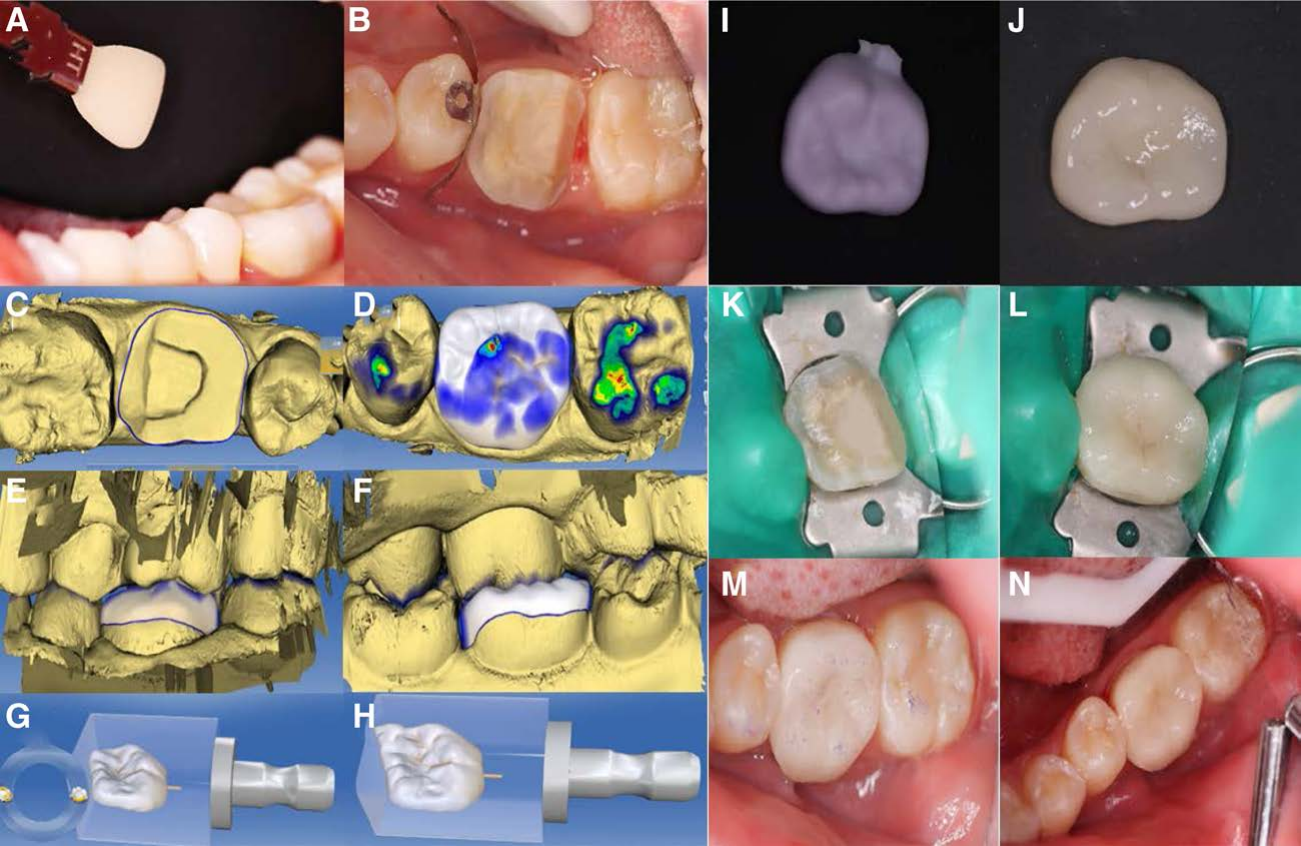
Crown restoration of tooth 36 at the 6-month follow-up visit. (A) Dental shade matching. (B–J) All-ceramic onlay preparation. (K–L) All-ceramic onlay installation. (M-N) Occlusal adjustment.

**Figure 12.**
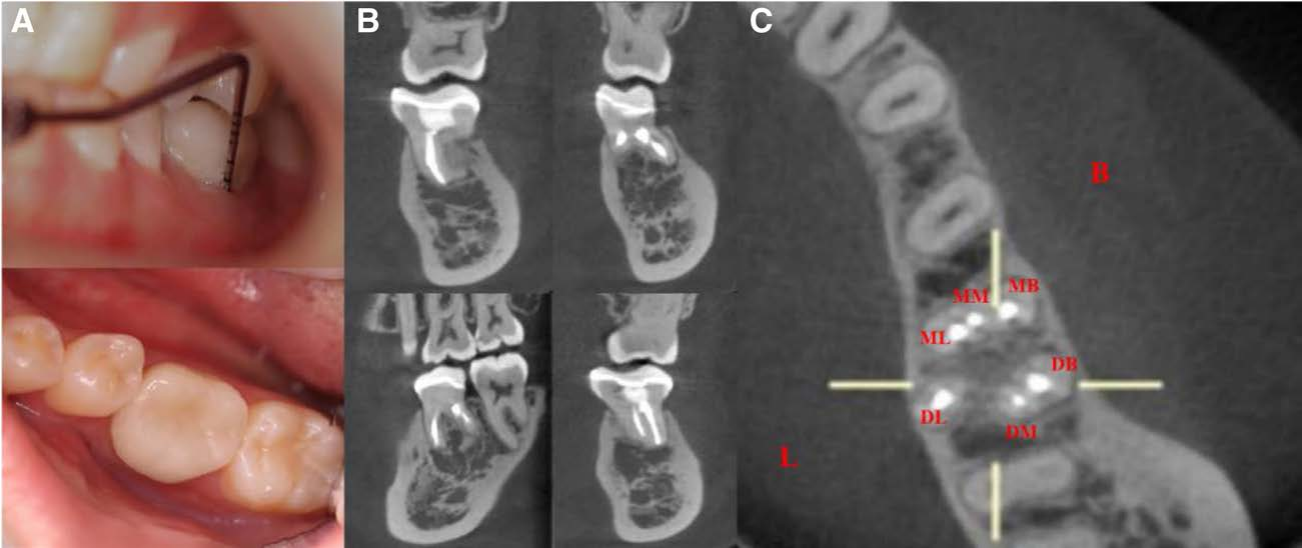
Intraoral photographs and CBCT images of tooth 36 at the one-year follow-up visit. (A) Intraoral photographs. (B onlay C) CBCT images. CBCT = cone-beam computed tomography.

At the 3-month follow-up visit, the patient was asymptomatic, and clinical examination indicated that there was no tooth mobility and tenderness to vertical percussion in tooth 36. The periodontal probing depth of the diseased tooth was within normal physiological limits, and no fistulae were observed in the soft tissue (Fig. 8). Additionally, CBCT evaluation revealed a clearly decreased apical lesion in tooth 36 (Fig. 9). The tooth continued to be free of symptoms at the 6-month follow-up visit, and it can be noted that further reduction of apical lesion was revealed in a CBCT analysis (Fig. 10). According to the patient’s wishes and excellent postoperative recovery, an all-ceramic onlay was provided as a permanent restoration to form a good seal and prevent tooth fracture (Fig. 11). Furthermore, complete resolution of the extensive apical lesion was achieved, as shown in CBCT images at the 1-year follow-up visit, confirming an overall successful treatment outcome (Fig. 12).

## 3. Discussion and Conclusion

The ultimate goal of root canal treatment is to eliminate microorganisms thoroughly in the entire pulp cavity via mechanical and chemical cleansing, while avoiding reinfection via complete root canal obturation with an inert filling material.^[14]^ It is well known that the mandibular first molar is the most common teeth to undergo root canal therapy in human dentition, which usually has a mesial root with 2 canals and a distal root with 1 or 2 canals. However, an increasing number of case reports have uncovered mandibular first molars with anatomical aberrances, such as lateral and accessory root canals. Several previous investigations have confirmed that the prevalence rate of additional canals in the mesial root of mandibular molars is reported to be 0.26% to 46.2%, while the incidence of a third distal canal ranges only from 0.2% to 3%. Therefore, special attention should be paid to root canal morphology and anatomical variance of mandibular first molars to avoid reinfection that extends along the root canal system into the apical area, which is one of the critical factors for endodontic treatment failure.^[15,16]^ The present report highlights an extremely rare case of an extensive periapical lesion in a mandibular first molar with 6 canals, including 3 mesial and 3 distal root canals. Currently, the precise molecular mechanisms involved in the development of periapical periodontitis are not completely understood. Nevertheless, it is generally agreed that adequate chemo-mechanical elimination of the microbial biofilm within the root canal system is the most vital factor responsible for achieving favorable treatment outcomes. Current treatment options available for large periapical lesions include root canal treatment, various surgical managements, or a combination of these options.

Root canal therapy was chosen in the present case as an initial treatment option for extensive apical lesions of tooth 36, instead of tooth extraction and periapical surgery. On the 1 hand, the treatment plan was formulated in accordance with a strong patient preference for preservation of the involved tooth, and the young patient age contributed to a favorable functional recovery. On the other hand, several studies have shown significant therapeutic advantages and clinical benefits of root canal treatment in cases of large periapical lesions having a direct connection with the root canal system.^[4–7]^ These findings appeared to be mostly associated with root canal infection, which could lead to the development of extensive apical inflammation through direct communication between the root canal system and periapical tissue. Successful root canal therapy completely eradicated pathogenic microorganisms inside the root canal to cut off the source of infection in these cases, thus achieving significant therapeutic efficacy. Additionally, nonsurgical and less invasive treatment options also provide several clinical benefits for patients with large periapical inflammation, including minimum psychological trauma and greater patient acceptance.

In the present case, follow-up visits were scheduled 3 months, 6 months and 1-year following after root canal treatment. Clinical examination and CBCT analyses demonstrated that the treated tooth remained symptom-free, and the apical damage was gradually reduced in size and radiolucency during 1-year follow-up period without any intervention, indicating a satisfactory prognosis for root canal therapy. Gratifyingly, the periradicular lesion spreading to the mesial root of tooth 37 was also completely resolved, as shown on postoperative CBCT images. In addition, calcium deposition within the 2 accessory canals in the mesial and distal roots, due to inflammatory stimuli, hampered root canal preparation, whereas there was no significant influence on the therapeutic effect of endodontic treatment. From a clinical standpoint, it may seem that the excellent treatment outcome in the present case was largely related to complete disinfection of the root canal system via successful root canal therapy and several physiological advantages afforded by the young patient age, such as ample periapical fibroblasts and osteoblasts, rich lymphatic drainage, abundant blood supply, root canal morphology, and dentinal structure.^[17]^ In addition, accurate information on the complex internal root morphology was readily available and accessible under microscope and CBCT image guidance, which provided us with the most appropriate approach to detect anatomical variations in tooth 36 with 6 canals.^[18–20]^ However, it is worth noting that only by accurate interpretation of CBCT images and oral examination can we detect the presence or absence of the third mesial and distal canals in the diseased tooth before and during root canal treatment.

This clinical report represents an extremely rare case of an extensive periapical lesion in a mandibular first molar with 6 canals, including 3 mesial root canals and 3 distal root canals, which reinforces the fact that a comprehensive understanding of not only the normal anatomy but also atypical anatomic variations within the root canal system should be developed to ensure successful endodontic treatment in dental clinical practice. Therefore, multi-angle CBCT images combined with close clinical inspection of the dental pulp cavity at high-magnification are indispensable when dealing with teeth with a high incidence of additional canals. Furthermore, the treatment outcome also confirms previous reports demonstrating the potential advantages of nonsurgical endodontic treatment for clinical implementation in cases of large apical lesions with a direct connection to the root canal system. However, further case reports and studies are recommended to clarify the precise influence of nonsurgical root canal treatment on extensive apical inflammation.

## Author contributions

**Data curation:** Xin Li.

**Investigation:** Shuyu Sun.

**Validation:** Tengyi Zheng.

**Writing – original draft:** Xin Li, Tengyi Zheng.

**Writing – review & editing:** Tengyi Zheng.
